# Acute Pain Management at the Intersection of Primary and Secondary Care: Insights from Recent Literature

**DOI:** 10.3390/jcm14217732

**Published:** 2025-10-31

**Authors:** Shouq S. AlGhamdi, Dalia M. Aljohani, Rosalind Adam, Patrice Forget

**Affiliations:** 1 Department of Anesthesia Technology, Prince Sultan Military College of Health Sciences, Dammam 34313, Saudi Arabia; shouq.alghamdi@hotmail.com (S.S.A.); johani.dalia@gmail.com (D.M.A.); 2 Epidemiology Group, Institute of Applied Health Sciences, University of Aberdeen, Health Sciences Building, Foresterhill, Aberdeen AB25 2ZD, UK; 3 Academic Primary Care, Institute of Applied Health Sciences, School of Medicine, Medical Sciences and Nutrition, University of Aberdeen, Aberdeen AB25 2ZD, UK; rosalindadam@abdn.ac.uk; 4 Pain and Opioids after Surgery (PANDOS) European Society of Anaesthesiology and Intensive Care (ESAIC) Research Group, 1000 Brussels, Belgium; 5 Department of Anaesthesia, NHS Grampian, Aberdeen AB25 2ZD, UK; 6 IMAGINE UR UM 103, Anesthesia Critical Care, Emergency and Pain Medicine Division, Nîmes University Hospital, Montpellier University, 30900 Nîmes, France

**Keywords:** acute pain, pain management, primary care, secondary care

## Abstract

Acute pain is a significant clinical challenge in primary and secondary care. If inadequately managed, acute pain is a major risk factor for the development of chronic postsurgical pain and persistent postoperative opioid use. In this perspective article, we offer the authors’ viewpoints informed by selective literature with the aim of helping identify avenues to improve the quality and safety of acute pain management. Current evidence in the quality and safety of pain management shows that the role of primary care staff in education, follow-up, and tapering remains largely unaddressed, while patient perspectives and experiences reveal gaps in communication, stigma, and trust. Screening tools, guidelines, and strategies to reduce variations in the prescription and use of opioids remain inconsistently developed. In conclusion, if pain is multifaceted and should be approached in a multidisciplinary way, strong integration between primary and secondary care is essential. Communication with primary care, tapering, or referral should be facilitated. Patient experience should receive more attention, and future studies should focus on implementing guidelines and multidisciplinary guidance, as well as providing continuous feedback to clinicians on their practice and outcomes.

## 1. Introduction

Acute pain is a common and often distressing experience. It occurs suddenly as a result of being exposed to any type of injury and lasts from minutes to less than three months [1,2]. Acute pain, particularly in postoperative and post-traumatic settings, is a significant clinical challenge with substantial patient and healthcare system implications. Inadequately controlled acute pain can induce or prolong work absenteeism and impede postoperative and post-trauma recovery, prolong hospital stays, and increase healthcare costs. Furthermore, inadequately managed acute pain is a major risk factor for the development of chronic postsurgical pain (CPSP) and persistent postoperative opioid use (PPOU) [3]. Epidemiological data indicate that CPSP and PPOU affect a considerable proportion of patients, with incidence rates varying based on the type of surgery and patient population [4]. Research identifies system-level and patient-level risk factors for PPOU. For instance, PPOU is strongly associated with preoperative opioid use, longer prescription durations (rather than dosage), and certain surgery types such as knee arthroplasty, open thoracic, and lumber fusion procedures [5]. Effective acute pain management is crucial not only for immediate patient comfort but also for preventing long-term complications and promoting optimal recovery.

Despite clear evidence about the importance of holistic post-operative pain management, the roles of primary and secondary care are not well understood. There is consensus that improved communication, interaction, and collaboration between primary and secondary care could improve acute pain management [6]. The goal of this perspective article is to discuss recent developments and literature to help identify how reallocating resources may help improve outcomes in the context of acute pain management.

## 2. Methods

This article is presented as a perspective rather than a comprehensive review. It offers authors’ views informed by selected literature on the role of primary care in acute pain management. Two databases (Ovid MEDLINE and Embase) were searched from January 2022 to 2025 with the medical subject heading terms “pain management” and “primary care”. The timeframe limitation was selected to allow the focus on the most recent publications concerning acute pain management. Articles discussing pain management in other care settings were excluded.

## 3. From Secondary to Primary Care, and Vice Versa

Multidisciplinary approaches to pain management, involving general practitioners (GP), anaesthetists, physiotherapists, psychiatrists, pharmacists, and specialist pain nurse practitioners, are well established across care settings [7]. This collaborative way has been mainly proposed for chronic pain, while such approaches have been less consistently implemented in the acute or perioperative setting, despite the potential advantages of a holistic, biopsychosocial model. Acute postoperative pain can be severe, sometimes requiring coordinated input from multiple disciplines, and persistent pain necessitates re-evaluation to exclude surgical complications and, if necessary, referral to a pain specialist [8]. Importantly, psychological support is a critical element of multidisciplinary care, yet it is often undervalued in acute pain management. Patient satisfaction and adherence to treatment also rely on patient-centred communication, highlighting the importance of shared knowledge and collaboration across the care pathway [9,10]. A German study revealed that GPs valued multidisciplinary cooperation, especially when dealing with chronic or complicated pain. When primary care treatment reached its limits, multimodal pain therapy and non-pharmacological approaches were considered useful alternatives. Some GPs were concerned about the overprescription of opioids, whereas others actively directed patients to pain specialists or inpatient programs [11].

A recent Norwegian study evaluated the feasibility of a tailored, multidisciplinary intervention for chronic pain [12]. The intervention consisted of a general course on pain management and a customised course for specific patient symptoms, including sleep, a guided course on physical activity, nutrition, depression, stress, body-mind activity, anxiety, and life skills. At the initial consultation, expectations and treatment goals were clarified, and patients confirmed their readiness to participate. Participants went through the general course, followed by a consultation to identify their need for a personalised plan, which took place prior to the second part of the intervention. Seventy participants were recruited through GPs and an outpatient orthopaedic clinic; 81% completed the generic course, meanwhile 61% of participants completed the whole course. Overall, the co-developed intervention was feasible to implement within municipal healthcare services and deemed acceptable by the feasibility study participants, as the majority of participants, 98%, reported that they would recommend the intervention to others.

## 4. Patient Perspectives on the Role of Primary Care in Prolonged Opioid Use

### 4.1. Gaps in Patient Education and Follow-Up

Existing literature highlights gaps in patient education and follow-up when opioids are prescribed in primary care [13,14]. For example, in a Dutch primary care setting [15], the majority of patients reported not receiving adequate information about the side effects of prescribed analgesics and were only advised to read the leaflets, which patients often disregard. They recalled positive effects being re-informed (e.g., about how the medication would help them sleep). Some participants noted a lack of follow-up for repeat prescriptions. Meanwhile, participants tended to avoid regular visits when their GP denied a refill prescription. Participants did not recall receiving guidance about the potential drawbacks of prolonged opioid use or discussions about the possibility of tapering the opioids [15]. These findings highlight the important role of GPs in initiating, following up, and tapering opioid medications, and how clear communication and a multidisciplinary approach could reduce harm.

### 4.2. Building Trust and Patient-Centered Communication

Patient-centred communication is vital in pain management. Haverfield and colleagues conducted interviews to explore patients’ perspectives on such communication within pain management discussions in five primary care clinics in the United States of America (USA). They concluded that trust is rarely established during the first encounter with a physician but develops through repeated interactions because of the complexity of pain. This reciprocal interaction is particularly relevant to pain treatment. The pain experience may be impeded by related issues like experiencing previous abuse or self-harm, requiring extra time to address and report pain concerns [16]. Such findings should be well recognised by the multidisciplinary team managing the patient. An effective documentation system should be in place to summarise and make key patient ideas, concerns, and expectations accessible to the patient management team, if the patients consented, so they feel heard and do not have to repeat themselves with each new provider; this may support trust and improve overall care experiences.

### 4.3. Stigma Surrounding Opioid Use

The language clinicians use can shape patients’ experiences. In interviews with 40 patients at high risk of opioid use disorder about their interactions with GPs, the majority expressed that the term “addiction” evoked negative feelings. Instead, they suggested the word “dependence” to describe their condition, as it conveyed a more respectful and health-oriented framing when making health-related decisions. Beyond terminology, receiving information about opioid medications’ indications, risks, possible adverse events, and signs of misuse was important to the patients [17], suggesting that primary care providers can play a role not only in prescribing safely but also in ensuring conversations are framed in a way that fosters engagement rather than avoidance.

## 5. General Practitioners’ Role in Opioid-Related Behaviors

Melton et al. evaluated the role of GPs about their intentions to engage in behaviours or practices that could mitigate opioid-related morbidity and mortality. They used three patient scenarios, where they asked participants to indicate how many times they would participate in prevention behaviour across ten similar scenarios. Overall, physicians communicated opioids’ risks to patients (9.2 ± 2.2 out of 10 patients) but tended to avoid discussions about complicated topics related to opioid therapy such as “opioid misuse”. Screening tools/questionnaires to assess the risk of opioid misuse were infrequently used, with 60.4% of physicians indicating that they would not use the Current Opioid Misuse Measure (COMM) questionnaire to assess current misuse. The authors also noted that patients with an opioid use disorder were discharged from follow up with their GP, and naloxone was seldomly co-prescribed to reverse overdose [18]. This study highlights that interventions are required to engage GPs in a wider range of behaviours that could prevent opioid-related harm.

Interventions that directly target patients in primary care can complement efforts led by physicians. Does et al. conducted a pragmatic randomised controlled trial to test a patient activation intervention aimed at improving patients’ knowledge, skills, and confidence in managing chronic pain in primary care (USA) [19]. Although not tailored to individual patients, the intervention supported a range of pain types and severities from less severe conditions to those transitioning from acute to chronic pain. It served as a bridge to specialist treatment for those with higher pain severity. At 12 months, patient activation scores were comparable to those in the usual care group, but the intervention group showed significant improvements: reduced moderate to severe depression (OR = 0.40, 95% CI 0.18–0.87), increased overall health (OR = 3.14, 95% CI 1.64–6.01), greater use of patient portal resources (OR = 2.50), greater use of meditation (OR = 2.72) and exercise/physical therapy (OR = 2.24), and higher physical health scores (mean difference 1.63; 95% CI 0.27–2.98). Both study arms saw a decrease in opioid use over the study period, with no significant difference between the intervention and usual care groups. These results suggest that primary care interventions can enhance self-management and patient engagement, potentially influencing opioid-related behaviours.

The HEALing study [20] was a large cluster-randomised trial conducted in the USA that aimed to reduce the number of opioid-related deaths (ORDs). Despite important efforts to improve education of practitioners and support to patients, no difference was observed for the primary outcome (adjusted rate ratio of 0.91 for the ORDs, 95% confidence interval, 0.76 to 1.09; *p* = 0.30). The effect of the intervention did not differ according to patient characteristics. In this trial, intervention communities selected more than 800 strategies and implemented more than 600 strategies including overdose education, naloxone distribution, medications for opioid use disorder, and opioid safety. Interestingly, only 38% had been initiated by the start of the comparison year. While it is difficult to generalise to non-American contexts, one may argue the next steps could involve a closer collaboration between primary and secondary care, when appropriate, and more cohesive guidelines, multidisciplinary perspectives, and better implementation of pain management guidance, as these may be essential aspects to actually modify behaviours of practitioners, and, ultimately, improve clinical outcomes.

Arguably, primary care-based interventions to improve pain management and reduce patient harm could be optimised through closer collaboration between primary and secondary care, more cohesive guidelines, multidisciplinary teamwork, and better implementation of pain management guidance. This is supported by a recent systematic review of 22 studies [21], where educational (n = 12), guideline-focused (n = 3), multifaceted (n = 5), and pharmacist-led (n = 2) interventions demonstrated diverse effect sizes (small-medium n = 10, large n = 12). The interventions with the largest effect sizes included behavioural aspects, particularly regarding prescriptions (and guideline deviations); feedback to clinicians on outcomes was also effective.

## 6. General Practitioners’ Knowledge About Pain Management

One study surveyed GPs about their knowledge related to opioid drugs and their doses, management of specific patient groups, and the selection criteria for pain therapy. Eighty-four percent of the participants reported using WHO analgesic ladder drugs in various clinical situations. The correct conversion of opioid doses was only noted among 9% of the participants, while the correct assessment of clinical situations requiring opioids was noted among 27% of respondents [22]. These findings are consistent with other studies [23,24] which have highlighted knowledge gaps amongst GPs.

Interviews with GPs have shown that GPs use the WHO analgesic ladder to guide pain management decisions, along with their previous experience. The GPs rarely mentioned using other guidelines or searching for other scientific literature. Some mentioned adopting shared decision-making (SDM) with patients when the treatment decision had a low risk of patient harm. Other GPs preferred to make prescribing decisions without patient input [11]. GPs perceived patient involvement to be useful as a means of providing information and improving treatment adherence, rather than playing a key role in making decisions about the treatment [25].

Moreover, exploring GPs’ adherence to guidelines recommendations regarding opioid prescriptions revealed a lack of awareness of opioid guidelines [11]. The main deviations from guidelines were concerning opioid dosing and treatment plans. Some GPs appreciated the presence of tools to help in opioid dosage conversion to prevent dosing errors. They underlined the need for tools that support SDM, offer up-to-date information, and blend seamlessly into clinical work. All these points highlight the importance of guideline implementation efforts (like neuropathic pain screening and appropriate management, i.e., with alternatives to opioids, not recommended as first line but not covered by the WHO analgesic ladder, or with non-pharmacological options, not mentioned either). This also highlights the importance of developing opioid conversion tools, knowing that a universal tool, consensually validated, is still lacking.

## 7. Other Interventions That Could Improve Pain Management

Variations in the prescription and use of opioid drugs will be assessed in a multicentre prospective study, where the study investigators aim to recruit around 1500 opioid naïve patients experiencing acute pain. The study aims to assess pain patterns, opioid use, association between population diversity in terms of demographics, clinical, and emotional characteristics on outcome measures, care satisfaction, and handling unused opioid drugs throughout the treatment period. The protocol has been published and [26,27] full results are awaited [28].

The incorporation of digital components like a shared electronic case report form, the availability of teleconsultation for patients at risk of developing CPSP (between GPs and pain specialist), and the utilisation of an app that provides multimodal ways to manage pain including physical exercise, mindfulness training, and educational content, was evaluated as part of a cluster randomised trial conducted in Germany. This trial aimed to determine whether this comprehensive intervention could improve outcomes for patients at the acute or sub-acute stage of non-specific low back pain. The results significantly favoured the intervention group when it comes to pain reduction, all patient-reported outcome measures, and patient satisfaction, demonstrating that this innovative approach improves patient wellbeing by offering conservative methods like education, physical exercise, and psychological interventions that cause less strain on medical facilities, hence less expenses [29]. A systematic review and meta-analysis tested the effect of primary-care education on reassurance among patients with acute or sub-acute low back pain [30]. The paper revealed that education increases patient reassurance when compared to standard care with moderate to high level of evidence. The review also suggests that patients will be more assured if the educational intervention is delivered by a physician rather than by other healthcare providers. The effect of patient education on other outcome measures, like physical function and pain, was not assessed in this review.

In an attempt to examine the efficacy and safety of opioid prescription in cases of acute neck or low back pain, OPAL trial investigators recruited adult patients who presented to different primary care or ED sites in Sydney to receive either guideline recommendations in addition to an opioid drug or guideline recommendations plus a placebo. This trial was triple blinded with pain severity at 6 weeks as its primary outcome measure. A total of 347 patients were recruited and there was no significant difference between groups in terms of pain severity other than a small significant difference favouring the placebo group at week 52. Nevertheless, no significant adverse events were reported between both groups other than opioid-related adverse events within the opioid group. Given that opioid use raises global concerns like misuse, addiction, or overdose, the study findings recommended avoiding opioid prescriptions for patients present with acute neck or non-specific low back pain. Despite having missing data (almost 25% at primary endpoint), a sensitivity analysis was conducted and revealed that the findings would not be affected by this [31].

## 8. Summary

GPs play a pivotal role in opioid prescription; therefore, tools should be available to support their decisions/communication along with pain-related educational materials to keep them up-to-date on pain management practices. Poorly controlled pain results in negative drawbacks that can strain health services; therefore, mitigating these negative impacts in early stages is recommended, which necessitates collaboration across all concerned specialities for better patient outcomes. Patient information, especially regarding their pain management, should be available and accessible in different care settings. Table 1 provides an overview of the gaps highlighted in this paper, along with the corresponding recommended action and the responsible parties.

## 9. Limitations and Future Research

This perspective paper is based on selectively sampled literature and reflects key challenges and opportunities when it comes to managing acute pain in primary care settings. Much of the available evidence derives from studies conducted within specific health systems, and acute low back pain is over-represented, which may limit the generalisability. Nevertheless, the broad themes are relevant across many primary care settings.

PANDOS [32] is a large international observational study that aims to document perioperative opioid use and safety. The results of this study may identify baseline practice variations and help shape the future of pain management through the development of strategies to improve the delivery of care for surgical patients. Future research should include implementation trials to test the direct or indirect pain management strategies in primary care that reduce CPSP and PPOU while improving patient outcomes and the delivery of care. These implementation trials should be guided by implementation frameworks such as RE-AIM (Reach, Effectiveness, Adoption, Implementation, Maintenance) [33] or CFIR (Consolidated Framework for Implementation Research) [34], which emphasise context, adoption, and sustainability of interventions.

## 10. Patient and Public Involvement and Engagement (PPIE)

PPIE was incorporated through participation in the NHS Pain Community Appointment Day held in Aberdeen late 2024. During this event, patients living with persistent pain were invited to share their experience through interactive activities, including “paper roll” placement where participants could write responses to an open-ended question about their pain management goals. We asked people to write their pain management goals, but they spontaneously added their concerns. People stated that they wanted to be truly heard and that communication between health care providers and patients needs to be improved. People highlighted their own experiences of being prescribed “strong” medications that caused undesirable side effects of which they were not aware of or educated about. While this activity was not designed to inform this paper, the perspectives expressed during the event, particularly regarding communication, continuity, and shared decision-making, resonated strongly with the discussions presented in this paper.

## 11. Conclusions

Effective acute pain management is essential for patient recovery, wellbeing, and the prevention of CPSP and PPOU. This review highlights several key gaps and opportunities. Patients frequently report inadequate education, limited follow-up, and inconsistent communication about opioids in primary care, while physicians acknowledge knowledge gaps and the underuse of validated screening tools and guidelines. At the same time, emerging evidence shows that targeted multimodal behavioural interventions in primary care can improve outcomes, patient reassurance, and prescribing behaviours.

Pain is a complex phenomenon that benefits from collaborative approaches across relevant disciplines. Improving acute pain management requires coordinated efforts at multiple levels. At the clinical level, GPs play a crucial role in patient education at discharge, regular monitoring, and prompt tapering or referral to specialist care. At the interdisciplinary level, structured shared-care pathways between primary care, surgical teams, pain specialists, and pharmacists should be integrated into perioperative systems, supported by thorough documentation and communication strategies. At the health system level, predictive tools and safety measures to identify patients at risk of persistent pain or opioid misuse, along with clinician feedback mechanisms, could enable earlier intervention and more efficient resource utilisation.

Overall, the evidence indicates that effective acute pain management cannot rely solely on individual training but must be supported by integrated, multidisciplinary, and system-wide solutions. Embedding these strategies into routine care provides the best opportunity to reduce complications, optimise recovery, and enhance patient experiences across both primary and secondary care settings.

## Figures and Tables

**Table 1:** A summary table mapping the recommended actions to the identified gaps.

Identified Gap	Recommended Action	Responsible Party
Poor communication/documentation	Standardised discharge summary with analgesia tapering plan with access guaranteed to all concerned parties Identify high-risk patient for the development of CPSP, and establish a proper postoperative follow-up plan Encourage professionals to engage patients in their care plan Early flag of patient with ongoing postoperative pain for referral back to surgical team for re-assessment and exclusion of any surgical complications	Surgical team, GP and pharmacist
Lack of monitoring	Use of prescription programs to track ongoing opioid use Establish a follow-up plan to assess the function and tapering process Provide patients with a hotline service for more information and help after discharge	GP and pharmacist
Knowledge about pain management (GP)	Integrate pain and stewardship training into CPD Provide decision-support tools/checklists	Professional bodies, hospital and opioid stewardship champion.
Limited patient education	Teach-back method to ensure patient understanding of pain relief options (pharmacological and non-pharmacological	Pain team, GP, and pharmacist

## Abbreviations

The following abbreviations are used in this manuscript:

CPSP	Chronic postsurgical pain
PPOU	Persistent postoperative opioid use
COMM	Current Opioid Misuse Measure
ORDs	Opioid-related deaths
GPs	General practitioners
WHO	World Health Organization
SDM	Shared decision-making
OPAL	Opioid analgesia for acute low back pain and neck pain
PPIE	Patient and Public Involvement and Engagement

## References

[1] The International Association for the Study of Pain What is Acute Pain?.

[2] International Classification of Diseases 11th Acute Pain Definition 2025. https://icd.who.int/en/.

[3] Deumens R., Steyaert A., Forget P., Schubert M., Lavand’homme P., Hermans E., De Kock M. (2013). Prevention of chronic postoperative pain: Cellular, molecular, and clinical insights for mechanism-based treatment approaches. Prog. Neurobiol..

[4] Rosenberger D.C., Pogatzki-Zahn E.M. (2022). Chronic post-surgical pain—Update on incidence, risk factors and preventive treatment options. BJA Educ..

[5] Adams T.J., Aljohani D.M., Forget P. (2023). Perioperative opioids: A narrative review contextualising new avenues to improve prescribing. Br. J. Anaesth..

[6] Forget P., Patullo C., Hill D., Ambekar A., Baldacchino A., Cata J., Chetty S., Cox F.J., de Boer H.D., Dinwoodie K. (2022). System-level policies on appropriate opioid use, a multi-stakeholder consensus. BMC Health Serv. Res..

[7] Staudt M.D. (2022). The Multidisciplinary Team in Pain Management. Neurosurg. Clin. N. Am..

[8] El-Boghdadly K., Levy N.A., Fawcett W.J., Knaggs R.D., Laycock H., Baird E., Cox F.J., Eardley W., Kemp H., Malpus Z. (2024). Peri-operative pain management in adults: A multidisciplinary consensus statement from the Association of Anaesthetists and the British Pain Society. Anaesthesia.

[9] McCormack L.A., Treiman K., Rupert D., Williams-Piehota P., Nadler E., Arora N.K., Lawrence W., Street R.L. (2011). Measuring patient-centered communication in cancer care: A literature review and the development of a systematic approach. Soc. Sci. Med..

[10] Robinson J.H., Callister L.C., Berry J.A., Dearing K.A. (2008). Patient-centered care and adherence: Definitions and applications to improve outcomes. J. Am. Acad. Nurse Pract..

[11] Kornder N., Hill V.J., Groffebert S.N., Becker A., Viniol A., Lindner N. (2025). General practitioners’ decision-making strategies in the pharmacological treatment of musculoskeletal pain: A qualitative interview study. Eur. J. Gen. Pract..

[12] Gjesdal K., Skurtveit S., Djuv A., Paulsen A., Sevild C., Lid T.G. (2025). A feasibility study of a co-developed, multidisciplinary, tailored intervention for chronic pain management in municipal healthcare services. Scand. J. Pain.

[13] Quanbeck A., Robinson J., Jacobson N., Li X., Hennessy-Garza R., Landeck J., Cohen A., Madden L., Pulvermacher A., Brown R. (2024). Strategies to Deimplement Opioid Prescribing in Primary Care: A Cluster Randomized Clinical Trial. JAMA Netw. Open.

[14] Huo S.A., Bruckner T., Das A.L., Xiong G., Marcovitz D., Neikrug A.B., McCarron R. (2024). Opioid prescriptions following behavioral health training among primary care providers. BMC Med. Educ..

[15] Davies L.E.M., Koerkamp E.A.W.J.-G., Koster E.S., Dalusong K.-J., Koch B., Schellekens A.F.A., Heringa M., Bouvy M.L. (2024). Patients’ perspectives about the role of primary healthcare providers in long-term opioid therapy: A qualitative study in Dutch primary care. Br. J. Gen. Pract..

[16] Haverfield M.C., Giannitrapani K., Timko C., Lorenz K. (2018). Patient-Centered Pain Management Communication from the Patient Perspective. J. Gen. Intern. Med..

[17] Olson A.W., Bucaloiu A., Allen C.I., Tusing L.D., Henzler-Buckingham H.A., Gregor C.M., A Freitag L., A Hooker S., Rossom R.C., I Solberg L. (2025). ‘Do they care?’: A qualitative examination of patient perspectives on primary care clinician communication related to opioids in the USA. BMJ Open.

[18] Melton T.C., Hagemeier N.E., Tudiver F.G., Foster K.N., Arnold J., Brooks B., Alamian P.A., Pack R.P. (2022). Primary care physicians’ opioid-related prevention behaviors and intentions: A descriptive analysis. J. Opioid Manag..

[19] Does M.B., Adams S.R., Kline-Simon A.H., Marino C., Charvat-Aguilar N., Weisner C.M., Rubinstein A.L., Ghadiali M., Cowan P., Young-Wolff K.C. (2024). A patient activation intervention in primary care for patients with chronic pain on long term opioid therapy: Results from a randomized control trial. BMC Health Serv. Res..

[20] HEALing Communities Study Consortium (2024). Community-based cluster-randomized trial to reduce opioid overdose deaths. N. Engl. J. Med..

[21] Bansal N., Armitage C.J., Hawkes R.E., Tinsley S., Ashcroft D.M., Chen L.-C. (2025). Decoding behaviour change techniques in opioid deprescribing strategies following major surgery: A systematic review of interventions to reduce postoperative opioid use. BMJ Qual. Saf..

[22] Biesiada A.M., Ciałkowska-Rysz A., Mastalerz-Migas A. (2024). Opioid Treatment in Primary Care: Knowledge and Practical Use of Opioid Therapy. Healthcare.

[23] Pérez-Pérez L., Cárdaba-García R.M., Mayo-Íscar A., Barrero-Santiago L., de la Nava-de Arriba J., Montero-Cuadrado F. (2025). A cross-sectional study on pain neurophysiology knowledge among Spanish primary healthcare professionals. Sci. Rep..

[24] Brooks E.M., Park A., Tu K., Yang W.-H.T., Tong S.T. (2025). Identifying Strategies to Improve Implementation of Integrative Pain Management in Primary Care. J. Am. Board. Fam. Med..

[25] Nørgaard B., Simonsen E., Skotte N.A., Marcussen M. (2025). General Practitioners’ Perceptions of Patient Involvement—An Interview Study. J. Eval. Clin. Pract..

[26] Jeffery M.M., Ahadpour M., Allen S., Araojo R., Bellolio F., Chang N., Ciaccio L., Emanuel L., Fillmore J., Gilbert G.H. (2022). Acute pain pathways: Protocol for a prospective cohort study. BMJ Open.

[27] ClinicalTrials Variation in Opioid Prescribing and Use for Acute Pain in Diverse Populations 2020. https://clinicaltrials.gov/study/NCT04509115.

[28] U.S. Food and Drug Administration Real-World Data to Assess Variation in Opioid Prescribing and Use for Acute Pain in Diverse Populations. FDAGov 2022. https://www.fda.gov/science-research/advancing-regulatory-science/real-world-data-assess-variation-opioid-prescribing-and-use-acute-pain-diverse-populations-09132022.

[29] Priebe J.A., Kerkemeyer L., Haas K.K., Achtert K., Moreno Sanchez L.F., Stockert P., Spannagl M., Wendlinger J., Thoma R., Jedamzik S.U. (2024). Medical App Treatment of Non-Specific Low Back Pain in the 12-month Cluster-Randomized Controlled Trial Rise-uP: Where Clinical Superiority Meets Cost Savings. J. Pain Res..

[30] Traeger A.C., Hübscher M., Henschke N., Moseley G.L., Lee H., McAuley J.H. (2015). Effect of Primary Care-Based Education on Reassurance in Patients with Acute Low Back Pain: Systematic Review and Meta-analysis. JAMA Intern. Med..

[31] Jones C.M.P., Day R.O., Koes B.W., Latimer J., Maher C.G., McLachlan A.J., Billot L., Shan S., Lin C.-W.C., McLachlan H. (2023). Opioid analgesia for acute low back pain and neck pain (the OPAL trial): A randomised placebo-controlled trial. Lancet.

[32] Forget P., Cotton S., Keir H., Campbell L., Onyeakazi U., Aljohani D., Almodibeg B., Jutila L., Hauser W., Rosenberger D. (2025). Pain and opioids after surgery (PANDOS): Protocol of a prospective, international, observational cohort study. Eur. J. Anaesthesiol. Intensive Care.

[33] Holtrop J.S., Estabrooks P.A., Gaglio B., Harden S.M., Kessler R.S., King D.K., Kwan B.M., Ory M.G., Rabin B.A., Shelton R.C. (2021). Understanding and applying the RE-AIM framework: Clarifications and resources. J. Clin. Transl. Sci..

[34] Damschroder L.J., Reardon C.M., Widerquist M.A.O., Lowery J. (2022). The updated Consolidated Framework for Implementation Research based on user feedback. Implement. Sci..

